# Supplementary material for: Multidrug Combinations against SARS-CoV-2 Using GS-441524 or Ivermectin with Molnupiravir and/or Nirmatrelvir in Reconstituted Human Nasal Airway Epithelia
Source: Pharmaceutics. 2024 Sep 27;16(10):1262. doi: 10.3390/pharmaceutics16101262 (PMC11510096; doi:10.3390/pharmaceutics16101262)
Supplement: Supplementary file 1 [file pharmaceutics-16-01262-s001.zip › pharmaceutics-3158613-supplementary.pdf]

a

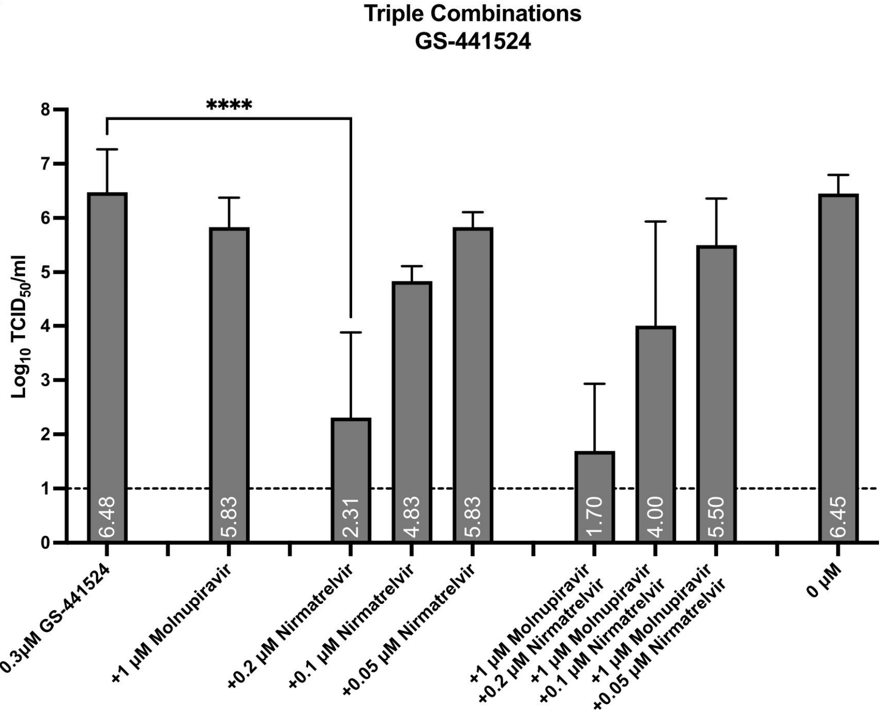

b

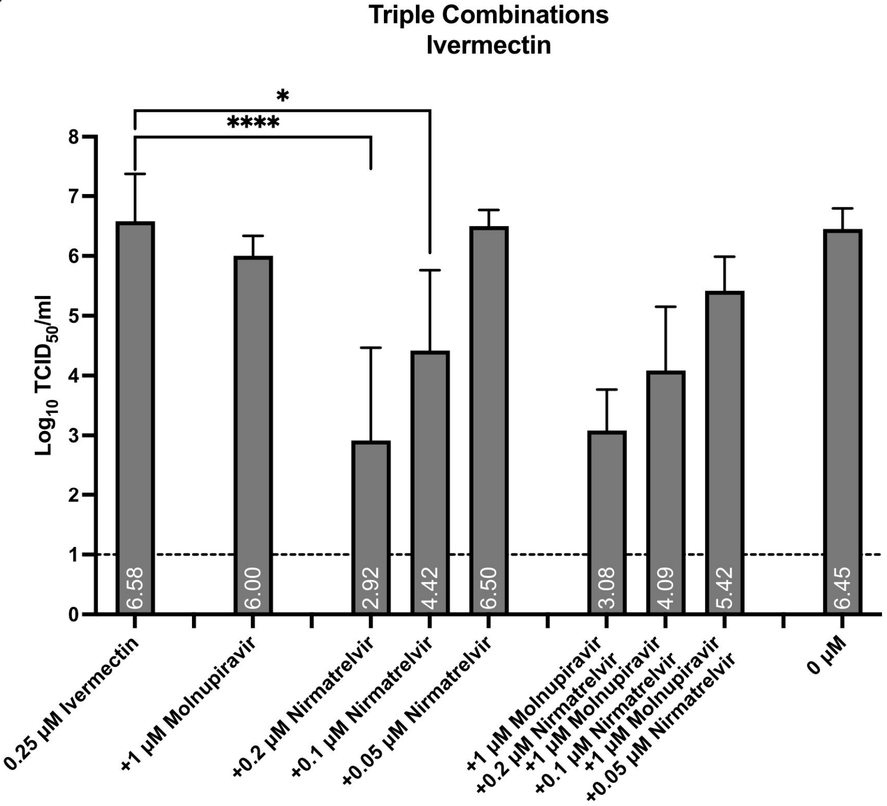

Figure S1. Lower concentrations of GS-441524 and ivermectin in combination with molnupiravir and/or nirmatrelvir. Triple combination treatment with suboptimal concentrations of (a) GS-441524 (0.3 μM, **** $p < 0.0001$) and (b) ivermectin (0.25 μM, * $p = 0.0233$, **** $p < 0.0001$). Data presented as mean ± standard deviation (SD), n = 3–7 cultures, from 2–4 independent experiments. Statistical significance was assessed using a non-matched one-way analysis of variance (ANOVA) with Tukey's multiple comparison test. Dashed line: lower limit of detection (LLOD), 1 $Log_{10}TCID_{50}$/mL.
